# Dynamics of transcription-coupled repair of cyclobutane pyrimidine dimers and (6-4) photoproducts in *Escherichia coli*

**DOI:** 10.1073/pnas.2416877121

**Published:** 2024-10-23

**Authors:** Ogϋn Adebali, Aziz Sancar, Christopher P. Selby

**Affiliations:** ^a^Molecular Biology, Genetics and Bioengineering Program, Faculty of Engineering and Natural Sciences, Sabanci University, Istanbul 34956, Türkiye; ^b^Department of Computational Science-Biological Sciences, Scientific and Technological Research Council of Türkiye (TUBITAK) Research Institute for Fundamental Sciences, Gebze 41470, Türkiye; ^c^Department of Biochemistry and Biophysics, University of North Carolina School of Medicine, Chapel Hill, NC 27599-7260

**Keywords:** transcription-coupled repair, MFD, molecular matchmaker, molecular cowcatcher, DNA excision repair

## Abstract

DNA repair processes modulate genotoxicity, mutagenesis, and adaption. Nucleotide excision repair removes bulky DNA damage, and in *Escherichia coli*, basal excision repair, carried out by UvrA, B, C, and D, with DNA PolI and DNA ligase, occurs genome-wide. In transcription-coupled repair (TCR), the Mfd protein targets template strand (TS) lesions that block RNA polymerase for accelerated repair by the basal repair enzymes. Accelerated repair is also seen with particular adducts. Notably, of the two major UV photoproducts, basal repair of (6-4) photoproducts [(6-4)PPs] is about 10× faster than repair of cyclobutane pyrimidine dimers (CPDs). To better understand repair prioritization in *E. coli*, we used XR-seq to measure TCR of UV photoproducts genome-wide. With CPDs, we found that TCR occurred at early time points, increased with transcription level, and was Mfd dependent; later, with completion of TS repair, nontranscribed strand (NTS) repair predominated. With (6-4)PP, when analyzing all genes, TCR was not observed; in fact, among the most highly transcribed genes, slightly more repair of (6-4)PPs in the NTS was evident. Thus, the very rapid basal repair of (6-4)PP in the NTS was faster than TCR of (6-4)PPs in the TS. Overall, TCR is of limited importance in (6-4)PP repair, and TCR of CPDs is limited to the TS of more highly transcribed genes. These results are consistent with the significant role of Mfd in mutagenesis and the modest effect of *mfd* deletion on UV survival and bear upon the response of *E. coli* to bulky DNA damage.

## Significance

Ultraviolet (UV) light induces principally cyclobutane pyrimidine dimers (CPDs) and (6-4) photoproducts [(6-4)PPs] in DNA. Both are excised from bacterial genomes by UvrABC excision nuclease. Due to relative helical distortion, (6-4)PPs are recognized and repaired about 10-fold faster than CPDs. A translocase, Mfd, targets RNA polymerase stalled at CPDs and accelerates transcribed strand repair up to the level of transcription-independent (6-4)PP repair. Mfd does not appreciably affect the repair rate of (6-4)PPs which are already efficiently repaired in both strands. Mfd is widespread in eubacteria, and these findings explain the significant role of Mfd in mutagenesis but its modest role in UV survival and provide insight into damage recognition in repair, mutagenesis, and development of drug resistance in pathogenic bacteria.

Ultraviolet light introduces mainly two types of DNA lesions, cyclobutane pyrimidine dimers (CPDs) and (6-4) pyrimidine-pyrimidone photoproducts [(6-4)PPs]. These lesions are removed by nucleotide excision repair in the form of 12 to 13 base oligomers in *Escherichia coli* and other prokaryotes and in the form of 26- to 27mers in humans and other eukaryotes (1, 2). The dual incision repair reaction is carried out by UvrA, UvrB, and UvrC proteins in *E. coli* and by six repair factors RPA, XPA, XPC, TFIIH, XPG, and XPF-ERCC1 in humans (3). Both in *E. coli* and in humans, it was discovered that transcription affects the rate of repair (4–7), specifically by stimulating the repair of CPDs in the transcribed strand (TS) such that the TS is repaired at 3- to 10-fold faster rate than the nontranscribed strand (NTS). Initially, these studies were carried out in a few select genes such as *DHFR* in humans and mice and the *lac* operon in *E. coli*. It was also discovered that transcription-coupled repair (TCR) was dependent on the CSB translocase in humans and rodents and the Mfd (Transcription Repair Coupling Factor = TRCF) translocase in *E. coli* and other eubacteria (8, 9).

In recent years, analyses of global repair and TCR were performed genome-wide in organisms ranging from *E. coli* and *Mycobacterium smegmatis* to Drosophila and mice and human cell lines (8–15). These studies confirmed and extended the previous single gene TCR studies but did not address in a quantitative manner the relationships between transcription rate and the TCR index (TS/NTS repair ratio). Here, we used wild-type and *mfd−* mutant *E. coli* cell lines, and we have determined the TCR index for CPD and (6-4)PP repair genome-wide and at single nucleotide resolution. We find that in wild-type cells, TCR of CPDs increases as a function of transcription level. After the TS is cleared of damage, there is greater repair of the NTS. For CPD repair in *mfd*− cells, the reverse trend is observed since initially, RNA polymerase stably stalled at TS adducts is known to inhibit their repair (16). Similarly, repair of (6-4)PP is initially greater in the NTS in *mfd*− cells. In wild-type cells, TCR of (6-4)PP repair is not observed when analyzing genes altogether, as previously suggested (17). Interestingly, in the most highly expressed genes of wild-type cells, there is slightly more repair of (6-4)PP in the NTS; thus, TCR of (6-4)PP is slower than global repair of (6-4)PP in the NTS. Since TCR of CPDs is limited to the most highly expressed genes, and TCR is a minor impediment to the more rapid global repair of (6-4)PPs, TCR is responsible for repairing a minority of UV damage in *E. coli*.

## Results

### Effect of Transcription Rate on TCR Index.

**Fig. 1.**
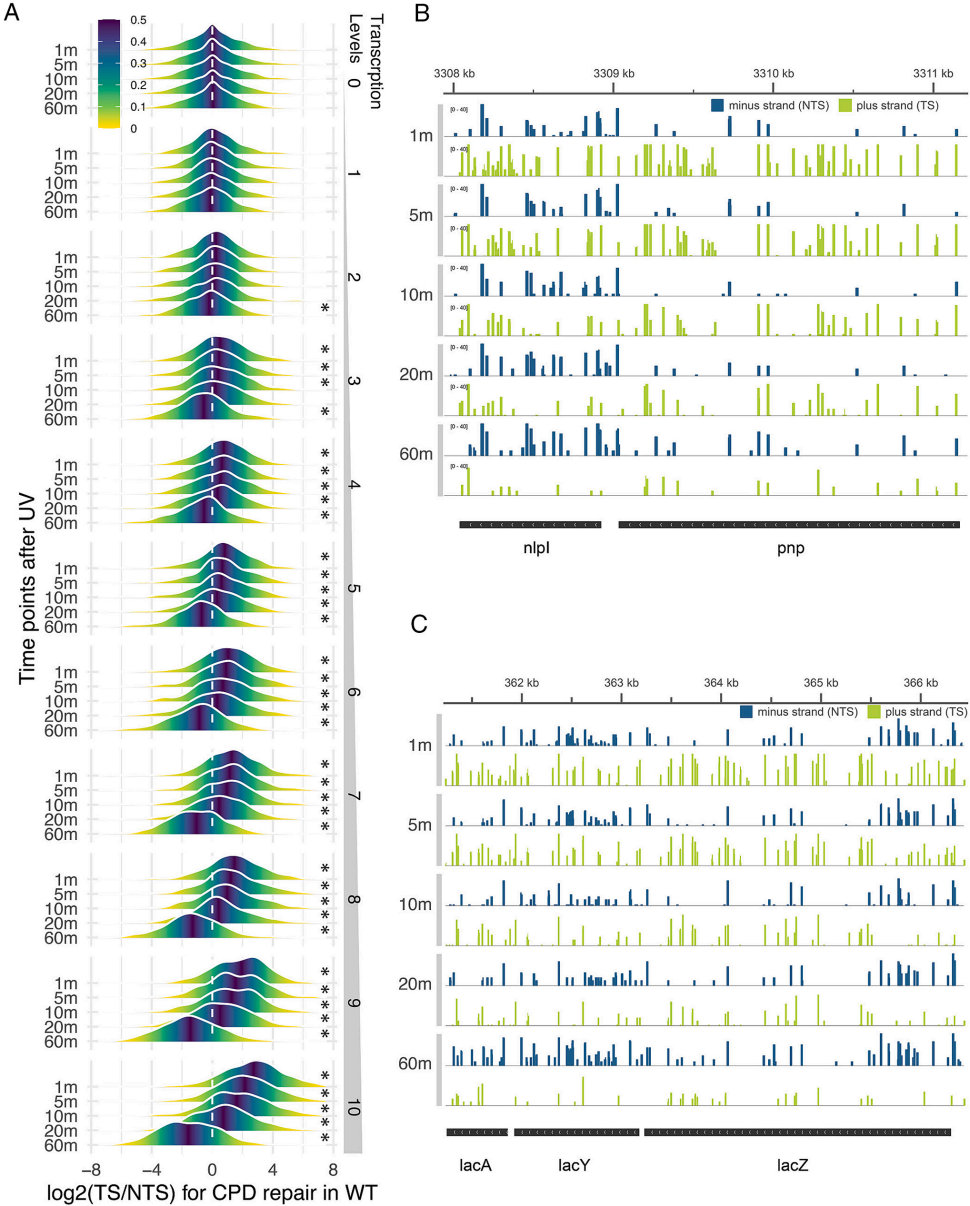
Time course of TCR of CPDs in wild-type cells. (*A*) Mountain plots showing the bias between transcribed (TS) and nontranscribed strand (NTS) repair. Each mountain represents 308 or 309 genes, in which binned log2(TS/NTS) values for each gene are plotted along the x-axis, with peak height (y-axis) the frequency of occurrence within each bin. 11 gene groups (0 to 10) are shown to the right with increasing expression levels from top to bottom. The five repair time points after UV exposure from 1 min to 60 min are shown to the left. All replicates are merged in this plot. Asterisks indicate results in which mean log2(TS/NTS) values are significantly different from zero at *P* < 0.05). (*B*) Screenshot showing CPD repair for two genes *nlpI* and *pnp. pnp* is known to be highly expressed. Relatively high repair on the plus, transcribed strand (green) particularly at the early time points shows robust TCR of the *pnp* gene. TS bias in repair weakens over time as the amount of CPDs on the TS is reduced, with a concomitant increase in NTS repair. The same trend is observed in the *nlpI* gene, which is in the same operon. Log2(TS/NTS) values for the *pnp* gene at the different time points are 7 (1 m), 5.7 (5 m), 5.5 (10 m), 5.2 (20 m), and 0.5 (60 m). (*C*) Screenshot of CPD repair in the *lac* operon. Cells were grown in IPTG to induce *lac* operon expression. The TS (green) is the plus strand. High TS bias is observed in CPD repair at early time points and is reversed over time. Log2(TS/NTS) values for the *lacZ* gene at the different time points are 3 (1 m), 2.6 (5 m), 1.2 (10 m), −2.1 (20 m), and −6 (60 m).

In order to characterize repair as a function of transcription, we first obtained transcription rates in *E. coli* by performing RNA-seq. Since all mRNAs have half-lives of ca. 5 min (18), we considered the level of a given mRNA relative to total mRNA to reflect its rate of transcription. We divided the annotated genes first into a category “0” with no or significant antisense transcription, and the rest were separated into deciles (=transcription levels) based upon transcription rate, with 1 being lowest and 10 highest. To assess repair, we used the XR-seq assay, in which excision repair products are isolated from cells, immunoprecipitated using anti-CPD or anti-(6-4)PP antibodies, ligated to adapters, repaired by photoreactivation, amplified by PCR, sequenced by next-generation sequencing, and sequencing reads are mapped to locate, at single nucleotide resolution, repair events throughout the genome (15, 19). In vivo, excision products are simultaneously generated and degraded; therefore, the resulting repair maps show a snapshot of repair at any given time point. XR-seq is sensitive even at early time points due to low background (20). Here, cells were irradiated with 100 J m^−2^ and sampled from 1 min to 60 min postirradiation, and then cells were harvested, and excision products were isolated, processed, and mapped. Fig. 1*A* shows “mountain plots” to illustrate strand bias in gene repair of CPDs in wild-type cells. In each mountain, 308 or 309 genes are plotted on the x-axis according to log2(TS/NTS) repair ratio with y-axis the frequency of occurrence. The different transcription levels/deciles are indicated along the right side and the different repair times are indicated to the left. (Note that all cells used in this study including the “wild type” had the genomic photolyase gene deleted to prevent artifacts due to photoreactivation.) Asterisks indicate data in which the median log2(TS/NTS) value for a given mountain is significantly different from 0 at *P* < 0.05. As apparent, in the 10th decile (*Bottom* panel), at the highest transcription level, the median TS/NTS ratio is about 8 (2^3^ = 8) at one min, and this median ratio decreases with time as the CPDs are depleted in the TS such that by 60 min postirradiation, repair in the NTS predominates. The other deciles show similar trends except with lower index values, and decile 0 genes with no, antisense, or modest transcription exhibit no change in repair ratio with time.

In Fig. 1 *B* and *C*, we show browser views of two operons *nlpI* (*pnp* -> *nlpI*) and *lac* to visually illustrate the change in the TCR index with time in these two highly expressed transcriptional units.

### Effect of *mfd* Mutation on TCR Index.

**Fig. 2.**
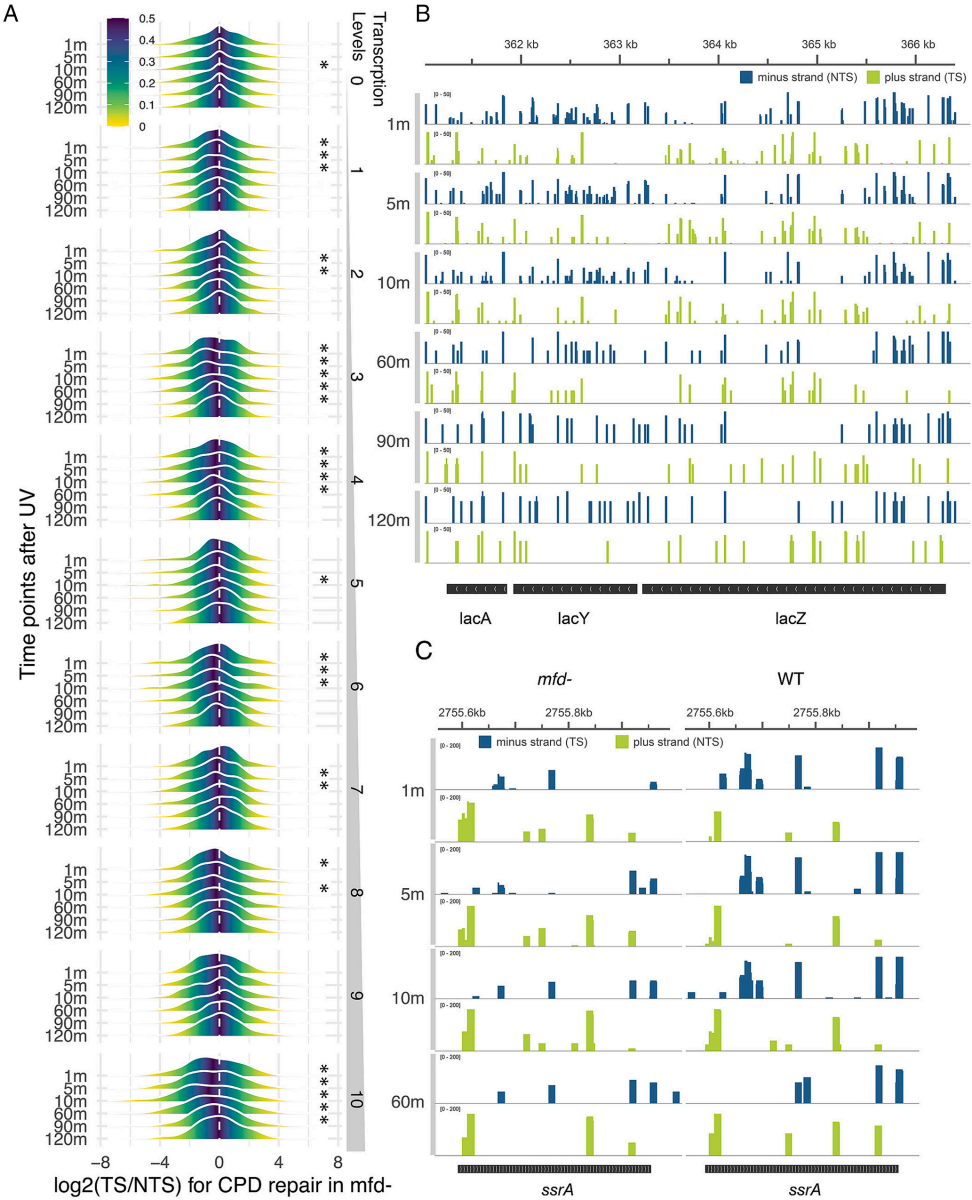
No TCR in an *mfd*-deficient cell line. (*A*) XR-seq data for *mfd−* cells are plotted as in Fig. 1. TCR, seen as favored TS repair at early time points in wild-type cells, is not observed in *mfd-* cells. Instead, at early time points and in highly expressed gene groups, higher repair of the NTS is observed, caused by inhibition of repair by RNA polymerase stalled at CPDs in the TS. All replicates are merged in this plot. (*B*) Screenshot of CPD repair in the *lac* operon in the *mfd-*deficient cell line. The plus strand (green) is the TS. Cells were grown in IPTG to induce *lac* operon expression. NTS bias is observed in CPD repair. Log2(TS/NTS) values for the *lac*Z gene at the different time points are −0.6 (5 m), −0.4 (10 m), −2.6 (60 m), −0.6 (90 m), and −0.04 (120 m). (*C*) TCR occurs independent of gene orientation. Screenshot comparing repair of the *ssrA* gene in *mfd−* and wild-type cells. Initially, there is more TS repair in wild-type cells and more NTS repair in *mfd−* cells. Thus, TCR is evident in this gene, in which the minus strand (blue) is the transcribed strand, unlike the lac operon in (*B*) and Fig. 1*C*, and the *pnp* gene in Fig. 1*B*, in which the plus strand is the transcribed strand. Log2 (TS/NTS) values for *ssrA* in WT cells are 3.7 (1 m), 3.4 (5 m), 3.03 (10 m), and −0.9 (60 m), and ratios for mfd− cells are −3.1 (1 m), −2.7 (5 m), −3.7 (10 m), and −3.8 (60 m).

Previous work has shown that the Mfd protein is essential for TCR in vivo (9–12, 21–32). Moreover, TCR has been reconstituted in a defined in vitro system consisting of UvrA, B, C, + RNA polymerase + Mfd + UV-irradiated DNA, and detailed structure–function studies have been conducted (21, 24, 31, 33–37). The absence of Mfd has a modest effect on UV survival but a major effect on UV-induced mutagenesis (38). Thus, the absence of Mfd is expected to dramatically affect the TCR index. With this background then, we performed XR-seq with an *E. coli mfd−* mutant and analyzed the data as was done with wild-type cells. Fig. 2*A* shows mountain plots of log2 (TS/NTS) for the 10 expression level deciles. There is a trend for a negative TCR index at times 1, 5, and 10 min, with most median values at these times significantly different from zero (meaning TS/NTS < 1.0). At the later time points, the trend is for higher medians which are mostly not significantly different from zero. These results are consistent with the fact that RNA polymerase blocked at damage sites inhibits repair of the transcription-blocking damages in the TS unless it is removed by Mfd or potentially Rho protein (39). However, removal by Rho in contrast to Mfd is not coupled to repair, and thus, the overall effect of the absence of Mfd is TS repair inhibition, albeit not as drastic as repair stimulation in the presence of Mfd. Thus, in *mfd−* cells, the preferential repair is modest and reversed from wild-type cells, with the NTS repaired more rapidly initially, followed by increased repair of the TS. Fig. 2*B* shows the effect of the absence of Mfd in a browser view of repair time course of the *lac* operon, which may be compared with *lac* operon repair in wild-type cells shown in Fig. 1*C*. We note that this switch of preferential strand repair in the absence of Mfd is not because of replication directionality. We observe (TS/NTS) TCR index >1.0 in the wild-type *E. coli ssrA* gene and its reversal in the *mfd−* background even though in *ssrA* the minus strand is the transcribed strand (Fig. 2*C*) as opposed to the *lac* operon where the plus strand is the transcribed strand (Figs. 1*C* and 2*B*).

### TCR of CPDs as a Function of Repair Time and Transcription Level.

**Fig. 3.**
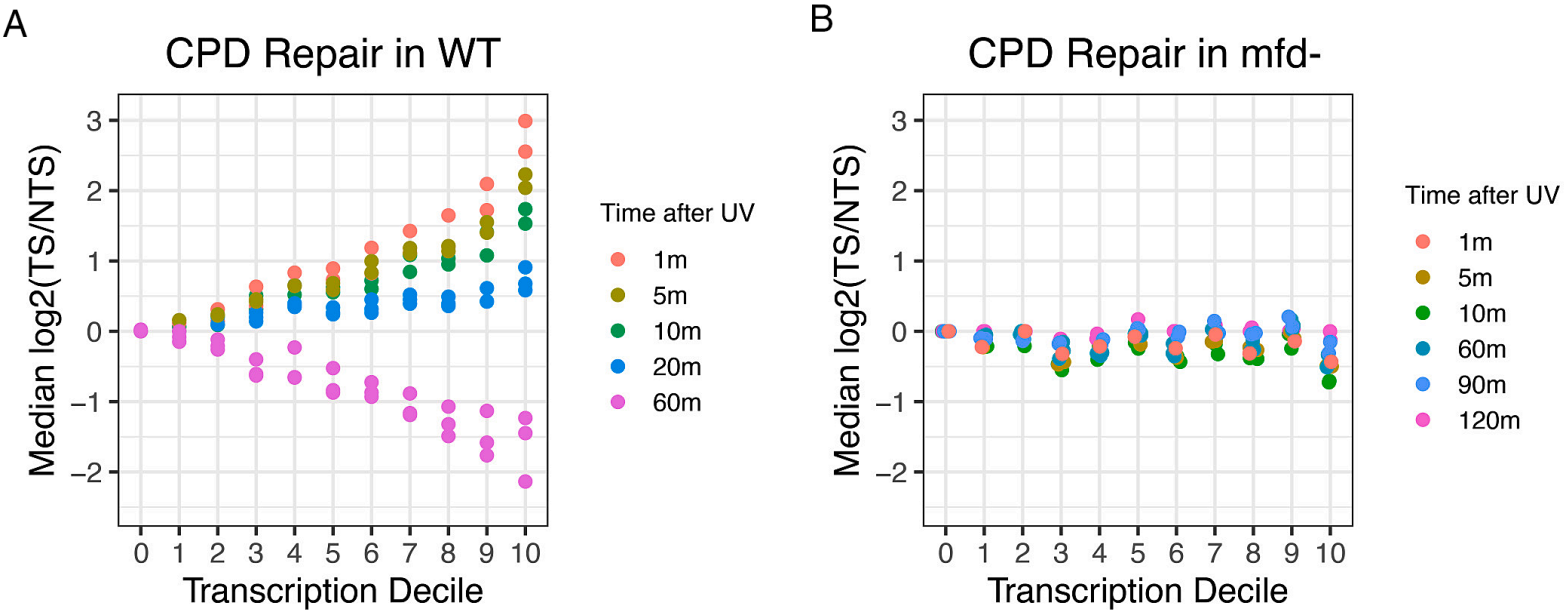
TCR of CPDs as a function of repair time and gene expression and dependence upon Mfd. (*A*) TCR in wild-type cells. The y-axis shows the median log2-transformed TS/NTS values for each gene group. The x-axis shows the 11 expression groups from low to high transcription. Each biological replicate is plotted. (*B*) Same as in *A*, in the *mfd−* cell line.

To more clearly compare TS and NTS repair of CPDs in wild-type and *mfd−* cells, we plotted the median TCR index at each repair time as a function of transcription decile. Fig. 3 *A* and *B* show that in decile 0, the repair rates of the NTS and TS in both cell lines are equal and do not change with time. Fig. 3*A* shows that for wild-type cells, the TCR index log2(TS/NTS) is greater than zero, highest at 1 min repair, and generally decreases with time, becoming < 0 at 60 min (red circles) for all deciles. In *mfd−* cells, Fig. 3*B*, a different trend is observed. At the early time points, log2(TS/NTS) ratios < 0 are common among deciles, and the trend reverses to log2(TS/NTS) closer to 0 or slightly positive at longer time points as the NTS is cleared of CPDs and repair switches to the TS. It is clear that in the absence of Mfd, TCR, the preferential repair of the TS as an initial response to damage, is abolished in genes in all classes of transcription rates.

### Role of TCR in (6-4) Photoproduct Repair.

(6-4)PPs are the second major DNA lesions induced by UV, constituting 10 to 20% of total UV photoproducts (7, 17). It was reported that rifampicin, which inhibits transcription significantly (ca. 50%), inhibits overall repair of CPDs but had no effect on (6-4)PP repair rate or extent. From this observation, it was concluded that (6-4)PPs are not subject to TCR (17). However, this global repair assay did not have the resolution to detect TCR of (6-4)PP if it existed. The (6-4)PP is a more efficient substrate for excision repair than CPD and hence its TCR index is expected to be small and, if it exists, only detectable with high-resolution assays. With these considerations then, we applied the XR-seq assay to measure (6-4)PP repair in wild-type cells.

**Fig. 4.**
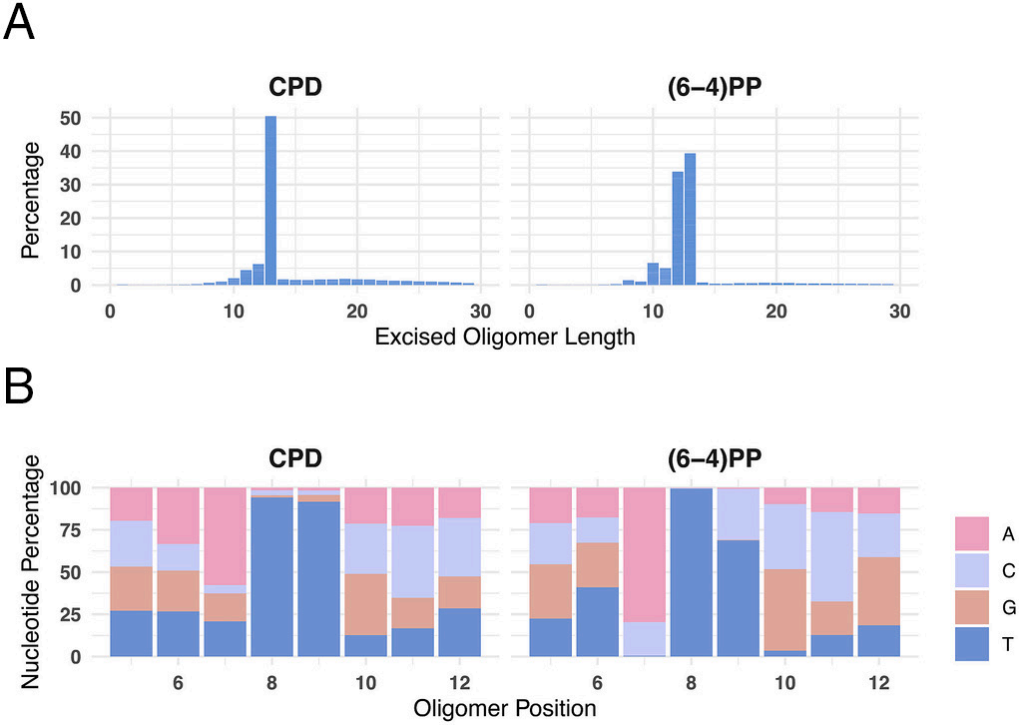
Size and composition of CPD and (6-4)PP repair products. (*A*) Plots of read length distribution frequency for CPD and (6-4)PP repair products. Although 13-mers are the most abundant in both, 12-nt (6-4)PPs are almost as abundant as 13-mers which is a distinct feature of (6-4)PP repair. (*B*) Nucleotide frequencies in excised oligomers. Results for 12-mers are shown, and nucleotides at positions 5 to 12 are shown. In UvrABC excision products, the damaged site is located at the 8th and 9th positions, which are enriched with dipyrimidines: principally TT for CPD and TT and TC for (6-4)PP. Position 1 corresponds to the 5′ end of excision products. The data plotted are in aggregated form from all time points.

In processing the sequencing data for analyses, we observed some unique aspects of (6-4) photoproduct repair in *E. coli* that we wish to mention here. First, for (6-4)PPs, the excised fragments are 12 and 13 nt in length as opposed to CPDs which are mostly removed in 13 nt long fragments (Fig. 4*A*). Second, while in *E. coli* and humans, the damaged residues in the (6-4)PP-containing excision product are preceded on the 5′ side predominantly by A or C in the XR-seq assay; in *E. coli,* A dominates (nearly 80%), as shown in Fig. 4*B*, while in humans, C dominates (nearly 75%) (15). In humans, AT content is 59% of the genome, while in *E. coli,* it is 49.6%. The small difference in AT content is unlikely to explain this preference, suggesting the reaction mechanism of the evolutionarily unrelated excision repair systems have different sequence preferences.

**Fig. 5.**
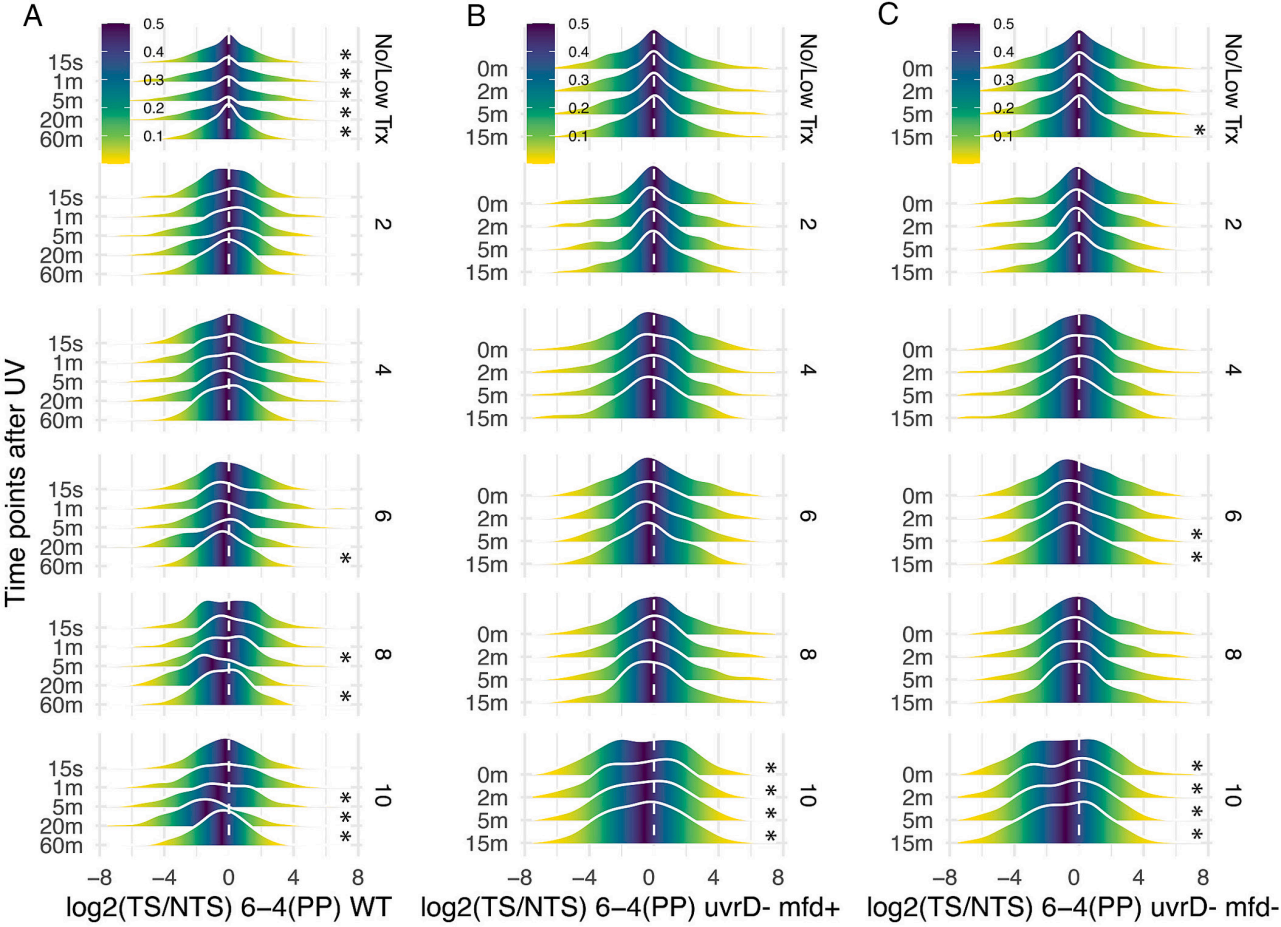
XR-seq analysis of (6-4)PP repair. (*A*) Mountain plots for (6-4)PP repair in wild-type cells from 15 s to 60 min after UV exposure. Only results from “No/Low Transcription” and even deciles are shown for brevity. Asterisks indicate results in which mean log2(TS/NTS) values are significantly different from zero at *P* < 0.05. (*B*) Same as in *A*, but in *uvrD*− *mfd+* cells. Also, in contrast to panel *A*, CPDs were eliminated by photoreactivation prior to excision repair. (*C*) Same as in *B*, but in *uvD− mfd−* cells.

The (6-4)PP XR-seq results for wild-type cells are shown with decile plots in Fig. 5*A*. The TCR index is not positive for any time point/decile. Median log2(TS/NTS) values become more negative with longer repair times, as apparent visually and by the fact that more median values are significantly different from zero at the later time points. This trend for preferential NTS repair could be a consequence of faster global repair of (6-4)PPs (in both strands) compared to TCR of (6-4)PP in the TS. However, it is unclear why, for the rapidly repaired (6-4)PP, such an effect would be observed at late time points.

The (6-4)PPs, although more efficiently repaired by the excision nuclease than CPDs, only constitute 10 to 20% of total UV lesions and hence their repair kinetics may be complicated by the presence of 5- to 10-fold more abundant CPDs. Hence, to overcome this problem, we eliminated CPDs by using cells transformed with a plasmid that encodes a CPD photolyase and then photoreactivating the UVC-irradiated *E. coli* before repair. Furthermore, we decided to analyze (6-4)PP repair in *mfd+* and *mfd− E. coli*, each in a *uvrD*− background since in the absence of DNA helicase II (=UvrD), the excision product remains stably bound to the genome and reliably recovered in experiments, and *uvrD−* cells demonstrate robust TCR of CPDs that occurs early in repair since catalytic turnover of Uvr subunits is retarded in the absence of UvrD (10–12). For these experiments, cells were maintained on ice during the UVC irradiation and subsequent UVA photoreactivation and then warmed to room temperature for 0 to 15 min to allow the repair of (6-4)PPs. Repair was stopped by placing tubes with cells in ice water. A 0-min repair time point (no warming of irradiated and photoreactivated *E. coli*) was conducted to detect repair occurring when cells were warmed incidentally during transfers and centrifugations prior to cell lysis.

The time-dependent decile plots are shown in Fig. 5*B* for *uvrD− mfd+* and Fig. 5*C* for *uvrD− mfd−* cell lines. As apparent from these plots, with both cell lines, among most deciles, the TS and NTS are repaired equally. However, there is clearly a trend for increased NTS repair at all time points in the highest transcription deciles.

**Fig. 6.**
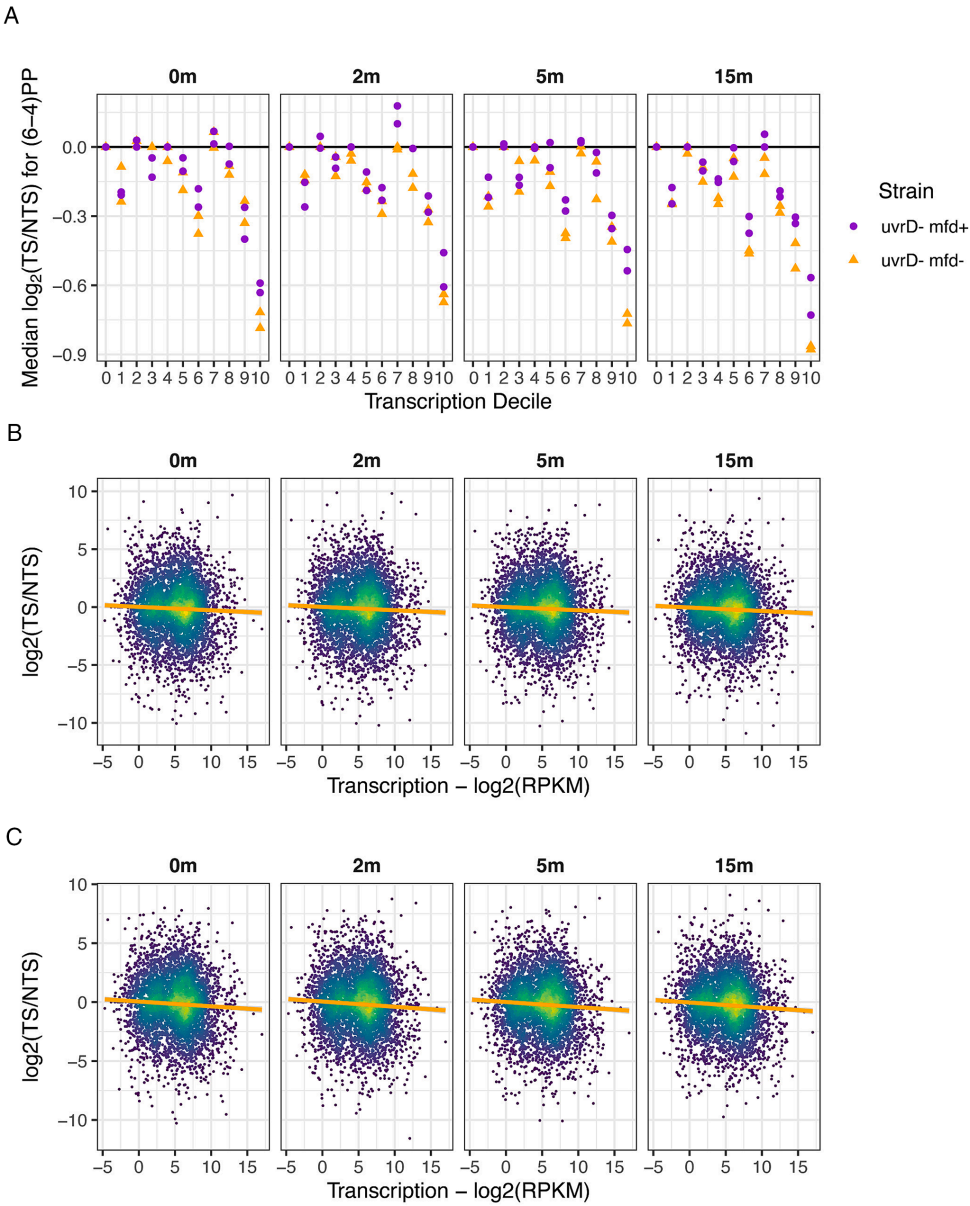
Quantification of TCR of (6-4)PPs in the presence and absence of Mfd. In these experiments, CPDs were removed by photoreactivation before undergoing excision repair. (*A*) Plots of median log2-transformed TS/NTS values (y-axis) as a function of transcription decile (x-axis) for two cell lines, *uvrD−* and *uvrD− mfd−*. All replicates are shown. Most values are below 0, showing that NTS repair is more favored than the TS. This asymmetry is more pronounced in the highly expressed genes, and in the absence of Mfd. In *mfd+* cells, Mfd removes the repair-inhibiting, stalled polymerase, which partially but not completely alleviates inhibition of TS repair because TCR is slower than global repair of (6-4)PPs. Values for median log2 (TS/NTS) are significantly different from zero at deciles 9 and 10 (*t* test *P* < 0.05) in both *uvrD− mfd−* and *uvrD− mfd+* cells. (*B*) Strand asymmetry as a function of transcription in u*vrD− mfd+* cells. The y-axis shows log2-transformed TS/NTS values, and the x-axis shows the log2-transformed RPKM values for transcription level. There is a weak negative correlation in each plot. (*C*) Same as in *B*, except the cells are *uvrD− mfd−*. A weak negative correlation is also seen with these data. The trend lines were plotted using geom_smooth option in ggplot with the “lm” model. Pearson correlation coefficient values range from −0.04 to −0.06, and all of the *P*-values for all time points in both strains are lower than 0.001.

To explain these results, we envision two ways in which repair of (6-4)PPs in the TS may be slowed. First, in *mfd−* cells, RNA polymerase inhibits repair of transcription-blocking (6-4)PPs, the same as it blocks repair of transcription blocking CPDs (16). In *mfd+* cells, the stalled polymerase is removed during TCR, alleviating TS repair inhibition to a degree, but the TCR of (6-4)PP that does occur is slower than global repair of (6-4)PP. In this scenario, preferential NTS repair will be greater in *mfd*− than in *mfd*+ cells. For a more quantitative evaluation, the XR-seq data were replotted in Fig. 6*A* which shows median log2(TS/NTS) ratios for all time points, deciles, and both strains. Clearly, at the 9th and 10th deciles, there is a trend for greater NTS repair in *mfd*− than in *mfd*+ cells, indicating that removal of the stalled polymerase during TCR in *mfd+* cells partially alleviates inhibition of (6-4)PP repair in the TS by RNA polymerase stalled at the (6-4)PP.

To assess the (6-4)PP repair genome-wide in *uvrD− mfd+* and *uvrD+ mfd−* cells, log2(TS/NTS) values were plotted as a function of transcription level for all genes in Fig. 6 *B* and *C*, respectively. The fitted lines illustrate modest but significant trends showing reduced TS/NTS ratios with higher transcription level in both *mfd*+ (Fig. 6*B*) and *mfd*− cells (Fig. 6*C*). These results are consistent with a prior finding of no TCR of (6-4)PPs in *E. coli* based on single gene measurements (17).

## Summary and Conclusions

This study confirms and extends previous work on TCR in *E. coli*, genome-wide and in a quantitative manner for the two major UV-induced DNA lesions. Our work leads us to make the following conclusions.

First, for CPDs which have a modest effect on DNA structure and are poorly recognized by the excision nuclease, the magnitude of TCR increases as a function of the transcription rate. Second, as measured by the rate of excision at early time points, excision products from the TS dominate, and at later time points when the majority of CPDs in the TS are removed, CPDs excised from the NTS gradually become majority. Third, TCR is absolutely dependent on Mfd protein which is the reason it is called Transcription-Repair Coupling Factor. In fact, in *mfd*− cells, the trend is for preferential transcription-dependent, NTS repair especially at early time points, in agreement with the finding that in vitro, RNA polymerase stalled at a CPD impedes excision repair of the transcription-blocking CPD. Fourth, for the (6-4) photoproduct, TCR is not detectable for *E. coli* genes overall. This finding is in agreement with the report showing that although rifampicin inhibits transcription and reduces the total rate of CPD repair by about 50%, it has no effect on the rate of repair of (6-4) photoproducts (17). This is because (6-4)PPs, in contrast to CPDs, induce considerable distortion to the DNA helix and are readily recognized by the excision repair enzyme and repaired approximately 10-fold faster than CPDs (40). In wild-type cells, this results in (6-4)PP being repaired at a faster rate in both strands than the rate of TCR of CPDs in the TS. In fact, it was also shown that when Uvr proteins were overproduced in cells or added in excess to in vitro reactions, even CPDs were repaired in a TCR-independent manner (17, 41). In addition, we note that a lack of TCR of (6-4)PP or only borderline TCR index is observed in humans, yeast, and Arabidopsis as well, consistent with the notion that the (6-4)PP is recognized about 10-fold more efficiently than CPDs by both the prokaryotic excision nuclease (40) and the eukaryotic type excision nuclease (42). Finally, we wish to point out the sequence preference of *E. coli* and human excision nuclease in removing the most common (6-4) photoproduct, T[6-4][T/C], preferentially in the C[T[T/C]] context in humans (15), while the *E. coli* excision nuclease exhibits 80% preference for A[T[T/C]] for (6-4) photoproducts. Thus, the distinct nature of (6-4)PP excision by eukaryotic and prokaryotic systems appears to be intrinsic to the recognition mode of these two nucleotide excision repair systems which even though they remove damage by dual incision, they are not evolutionarily related.

**Fig. 7.**
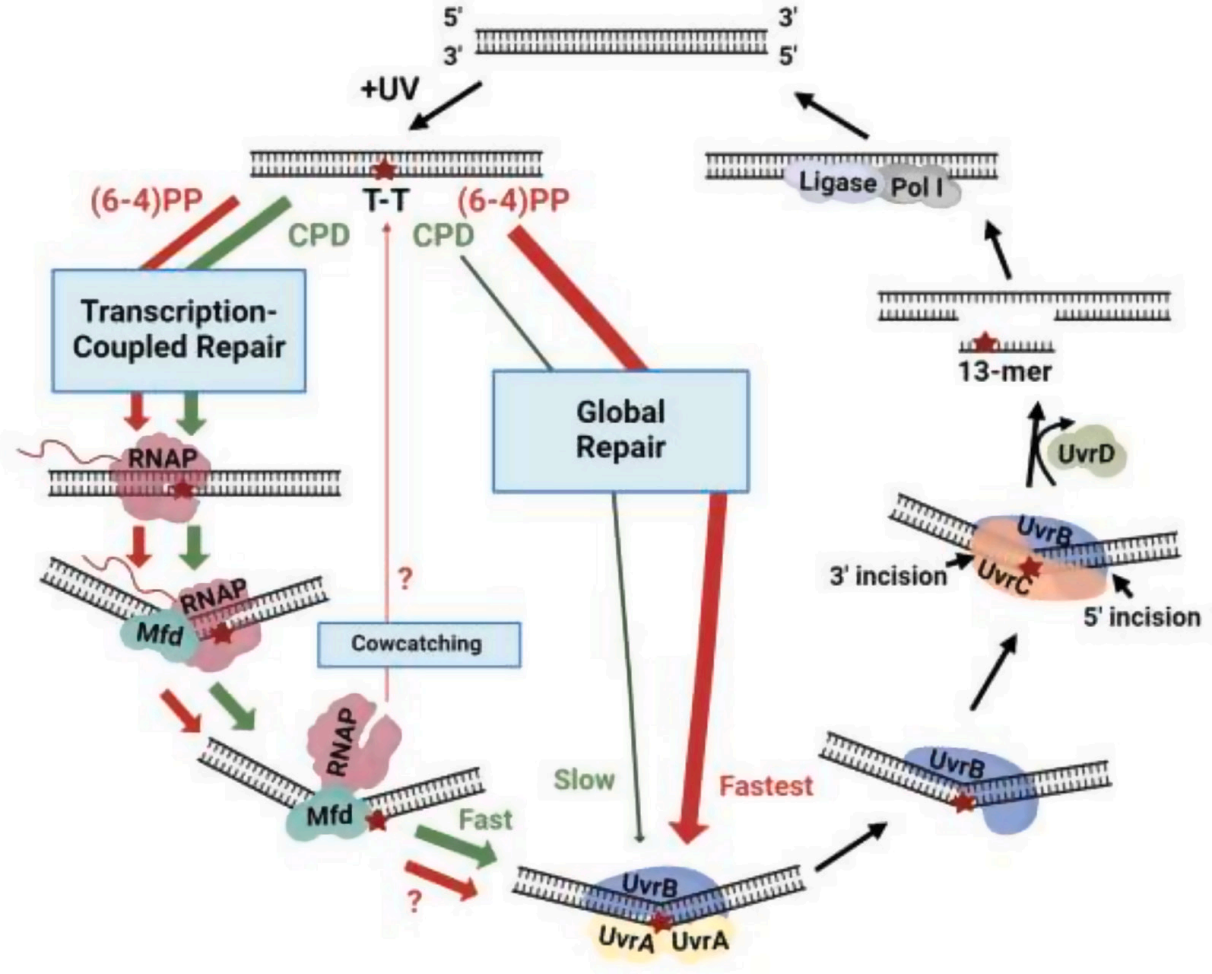
Excision repair in *E. coli*: global repair and TCR of the template strand of a strongly transcribed gene. At T-T and other dipyrimidine sites, absorption of UV light produces principally CPDs and (6-4)PPs. Both are bulky DNA adducts (indicated with a star). In global repair, UvrA functions as a bulky DNA damage sensor; it loads UvrB onto the DNA damage and dissociates. UvrC then interacts with the UvrB preincision complex, and dual incisions 5′ and 3′ to the damage are made in the damaged strand. UvrD promotes dissociation of the Uvr proteins and the damage-containing 12- to 13-nt oligomer and promotes synthesis of a repair patch by DNA PolI and DNA ligase. In TCR, damage in the transcribed but not the nontranscribed strand blocks elongation by RNA polymerase. The stalled polymerase and DNA immediately upstream of the polymerase are bound by the Mfd protein, a translocase which induces a bend in the DNA, and upon translocation dissociates the polymerase from the template, forming a DNA-Mfd-polymerase complex with release of the nascent RNA. Mfd in this conformation reveals a high-affinity binding site for UvrA, which is drawn to the damage site at an accelerated rate upon interaction with Mfd. UvrB is loaded onto the damage, Mfd and RNA polymerase are released from the DNA, and TCR is completed as in global repair. Arrow widths indicate relative repair rates. Based upon TS/NTS repair ratios, we find that TCR of (6-4)PPs is slower than global repair of (6-4)PPs. In fact, TCR of (6-4)PPs has not been directly demonstrated in vitro. Therefore, another mechanism may account for the slower TS repair of (6-4)PPs. Possibly after RNA polymerase stalls at (6-4)PPs, it is removed from the template by Mfd without repair (cowcatching), regenerating the duplex with the TS damage. Question marks indicate the uncertainty of these (6-4)PP repair pathways and their rates. After RNA polymerase encounters a CPD in the TS, Mfd and RNA polymerase are stably bound to the template, and TCR occurs following interaction with Uvr proteins.

A model illustrating global and TCR of UV damage is shown in Fig. 7. Wider arrows indicate faster repair rates. Compared to the slow global repair of CPDs, global repair of (6-4)PPs is about 10-fold faster, and TCR of CPDs is about eightfold faster. We find based upon TS/NTS ratios that global repair of (6-4)PP is also faster than TCR of (6-4)PPs. However, TCR of (6-4)PPs has not been directly demonstrated in vitro. Thus, it is hypothetically possible that if in fact (6-4)PPs do not undergo TCR, Mfd may remove RNA polymerase from (6-4)PPs without TCR (cowcatching), which would contribute to slower repair of (6-4)PPs in the TS. Cowcatching is unlikely at CPDs due to the stability of Mfd and RNA polymerase at CPDs during TCR (31). Thus, it is possible that Mfd functions as a molecular matchmaker for TCR of CPDs, but as a molecular cowcatcher for (6-4)PPs.

Our results demonstrate the value of XR-seq in detecting weak repair signals, notably the increased repair of (6-4)PP in the NTS of highly expressed genes, and the delayed repair of CPDs in the NTS in WT cells and the TS of *mfd−* cells. XR-seq is sensitive because it detects a discrete signal of excision products at nucleotide resolution with minimal background. In comparison, the classic assay successfully used to discover TCR (5, 6) measures the loss of T4 endonuclease sensitive sites (CPDs) at the gene level using Southern blots, as the difference in two values (time points) which may be small and variable. This has been problematic, as in the case of *uvrD*- cells, when measuring relatively weak repair signals (43). In contrast, repair and robust TCR is readily detected in *uvrD− E. coli* and *M. smegmatis* cells by XR-seq (10–12, 44).

Mfd’s role in TCR in *E. coli* has consequences in that deletion of *mfd* leads to a significant mutagenic phenotype and modest UV sensitivity (38). Enhanced UV mutagenesis in *mfd−* cells is consistent with error-prone replication occurring during the delay in repair in *mfd−* cells. Of note, it has been reported that in *mfd+* cells, UV damage in the more slowly repaired NTS of an active gene is more mutagenic, while in *mfd−* cells, damage in the more slowly repaired TS is more mutagenic (45).

Regarding UV lethality, for comparative purposes, the absence of excision repair conferred by defects in *uvrA*, *uvrB,* or *uvrC* produces strong UV sensitivity. The repair defect in *uvrD* mutants leads to substantial UV sensitivity, intermediate between *mfd* mutants and *uvrA*, *B*, and *C* mutants (46). UvrD functions in basal excision repair indirectly, in a downstream step following dual incision (Fig. 7) (47, 48). UvrD dissociates UvrB, UvrC, and the damaged oligonucleotide from postincision complexes, allowing catalytic cycles of repair (49). In the absence of UvrD, the level of excision is initially limited, with dual incision levels stoichiometric with the UvrB and C subunits, and repair synthesis and ligation inhibited. Thereafter, incision, repair synthesis, and ligation are all strongly delayed, awaiting noncatalytic turnover of UvrB, UvrC, and the excised oligonucleotide (50). The absence of Mfd, on the other hand, leads to a much more modest delay in the repair of only a fraction of the genome. Because of the way excision repair is prioritized in *E. coli*, most UV-damaged DNA is not repaired appreciably by TCR, including intergenic DNA, template strands of nontranscribed genes, much of the damage in the template strands of weakly and even moderately transcribed genes, nontranscribed strands of all genes, and for practical purposes, (6-4)PPs. In this context, and since repair-inhibiting RNA polymerase blocked at lesions in the template can be removed by Rho (albeit without TCR) (39), modest UV sensitivity of *mfd−* cells is consistent with the limited overall role of Mfd in excision repair.

The modest UV sensitivity conferred by *mfd* deletion may be considered a relatively weak selection pressure to account for the widespread presence of Mfd and TCR in eubacteria. To this point, it is relevant that Mfd has other roles in the cell (26, 51, 52). Mfd has been associated with elevated mutagenesis “spontaneously” produced in the absence of exogenously induced damage, which apparently enables *E. coli* to respond to adverse conditions including antibiotic treatment. The elevated mutagenesis could result from spurious excision repair in the absence of DNA damage, or possibly repair of endogenously produced damage, such as oxidative damage (53, 54). Mfd also is involved in stress responses, such as catabolite repression and toxin expression, by virtue of its involvement in gene expression (55–58). These roles are presumably related to the ability of Mfd to dissociate elongating RNAP, or reinitiate transcription of RNA polymerase stalled at pause sites or other impediments such as DNA-bound proteins or higher-order nucleic acid structures, as well as its involvement in recombination and R-loop formation (22, 26, 51, 52, 59). These roles may allow *E. coli* to tolerate or develop resistance to threats to survival.

## Materials and Methods

### Strains.

*E. coli* MG1655 strains MGP (*phr*−), MPdM (*phr− mfd*−), MPdD (*phr− uvrD*−), and MPdDdM (*phr− uvrD− mfd*−) have been described (11, 29). For (6-4)PP repair experiments, the latter two strains were transformed with pMalAnPL (Addgene), which expresses *Anacystis nidulans* CPD photolyase fused at the amino terminus to maltose binding protein.

### XR-Seq Assay.

For the XR-seq assay of MGP and MPdM, cultures were grown in LB (Miller) with or without IPTG (at 1 mM) to an OD_600_ of approximately 0.5 and cooled to room temperature by swirling the flask in ice water, and 15 mL aliquots were transferred to R-150 tissue culture dishes for irradiation. Cells were irradiated with mixing at room temperature with 100 J/m^2^ of UV-C (principally 254 nm) at a rate of approximately 2 W/m^2^. After predetermined repair time incubations at room temperature, plates were placed on ice water to stop repair. Repair time was included as irradiation time such that for irradiations lasting 50 s, dishes for one min repair time samples were put on ice water 10 s after the light was turned off. Survival under these conditions was on average 68% (MPD) and 50% (MPdM). 12 to 24 dishes containing 180 to 360 mL cells were irradiated at each time point, and assays of each time point were repeated, except the MPdM 1-min time point was done once. For assay of (6-4)PP repair in MPdD and MPdDdM cells, cultures of pMalAnPl-transformed cells at OD_600_ of about 0.5 were cooled on ice, and irradiated as above except on ice. Following irradiation, cells were transferred to ice-cold, clear centrifuge tubes and photoreactivated on ice water with black light illumination lasting 60 to 90 min. A glass plate was interposed between the black light and the cells during photoreactivation. For excision repair, tubes were then placed in room temperature water with mixing for predetermined repair times, then placed in ice water to stop repair. One to two plates of cells (15 to 30 mL) were used for each time point, and time points were repeated. Following repair, cultures were processed as described previously to obtain sequences of excision repair products produced from each population of irradiated cells (10, 11, 15).

For RNA-seq assays, cell lines noted were grown to an OD_600_ of about 0.5, cooled on ice, and cultures were processed as described in the Thermo Fisher PureLink Users Guide. Purified RNA samples were submitted to Novogen for sequencing.

## Data Analysis.

### Duplicate removal.

We employed unique molecular barcodes to eliminate PCR duplicate artifacts. The fastq files were sorted, and duplicates were removed using a custom script available in our repository.

### Adaptor trimming.

Post-PCR duplicate removal, we trimmed the 3′-end adaptor using cutadapt version 3.4, allowing flexibility in the unique molecular identifier adaptor region with the sequence: GGCTCAGTTCGTATGAGTGCCGNNNNNNNN. Reads without the expected adaptor were discarded using the “--discard-untrimmed” parameter.

### Quality control.

To ensure quality control of the XR-seq procedure, we calculated the read length distribution of excised oligomers and the nucleotide distribution of the most abundant excised products using custom scripts from our repository. We verified the presence of expected dipyrimidines at the expected damage sites.

### Alignment.

We aligned the reads to the complete *E. coli* reference genome (NC_0009133) using Bowtie2 (version 2.4.5) with the seed parameter “--seed 1.” The fasta file was downloaded from NCBI, and Bowtie2 index files were created using the bowtie2-build command. As demonstrated in our previous work (12), we focused on 12-mer oligomers.

### Screenshot visualization.

For screen visualizations, we used bigwig files. Aligned reads in bed files were separated into plus and minus strands using an awk command from our repository. These strand-separated bed files were converted to bedgraph format using “bedtools genomecov” with parameters -bg -scale N (where N denotes the total number of reads). The bedgraph files were then converted to bigwig format using ucsc-bedGraphToBigWig (version 377). Bedtools version 2.29.0 was used in this step, and the bigwig files were visualized using IGV.

### Obtaining read counts.

To account for differences in potential excision sites between genes and strands (TS and NTS), we conducted an excision repair simulation. We randomly generated N reads per sample (N being the total number of mapped reads) using a custom script (repository available). We identified “unrepairable sites” where no excised oligomer was observed. Only unique 12-mer potential excision sites observed in at least one biological replicate of any strain were used. We repeated random read generation for five times and used the median count value for each gene and its strand.

For both simulation and XR-Seq data, reads mapped to the TS and NTS of each gene were counted. All of the repair data shown in this paper are normalized to the randomly generated read counts to eliminate the effect of sequence context. The gene list was retrieved from a gtf file downloaded from NCBI and converted to a bed file using a custom script. To count repair events for each gene, we used bedtools intersect with -S and -s commands for TS and NTS, respectively.

### RNA sequencing analysis.

The adaptor trimming was performed with trimmomatic as originally applied with the following parameter: “TRAILING:3.” The alignment was performed with the STAR alignment tool (v 2.6.0). Transcript counts were calculated for sense and antisense strands separately for each gene using bedtools. For all wild-type repair data, the wild-type RNA-seq dataset was used (Figs. 1 and 3*A*). For all *mfd−* (single mutant) repair data, the *mfd−* RNA-seq dataset was used (Figs. 2 and 3*B*). For all *uvrD−* repair data, including *uvrD−/mfd−* double knockout, the *uvrD−* RNA-seq dataset was used (Figs. 4 *B* and *C* and 5). For both sense and antisense strands, RPKM normalization was applied. Because this study focuses on TCR, we aimed to eliminate the ambiguous transcription events close to the basal level as well as anti-sense transcription events, which may disrupt the strand asymmetry of TCR. If sense RPKM is lower than 3 or if sense/antisense ratio is lower than 2, we classify the genes as “no/low or ambiguous transcription” which corresponds to decile 0. We clustered the rest of the genes into deciles. The first decile has the lowest transcription and the tenth decile has the highest transcription level. In each decile, there are 308 or 309 genes.

### Statistical analysis.

Most of the samples show no normal distribution tested via the Shapiro test. Therefore, we used the Wilcoxon test to compare the means of two distributions as well as to test whether the TS/NTS ratio is different than 1. Asterisks in Figs. 1, 2, and 5 indicate where median log2(TS/NTS) values for all replicates are different from 0 at *P* < 0.05. In all cases, treatments were repeated (except one repeat only for *mfd−* cells, one min repair of CPDs).

### Computing resources and reproducibility.

The data analysis is fully reproducible, facilitated by a Snakemake workflow for scalability. All custom scripts, commands, and parameters for publicly available tools are in our repository. Data analyses were performed on computing clusters at Sabanci University, managed by the Slurm workload manager. The workflow can also run on any Linux-based server.

## Data Availability

The entire datasets of XR-seq and RNA-seq are deposited to the SRA database (PRJNA1155263) (60). The codes are available at https://github.com/adebali/bacnerumi.
